# Prevalence of ST1049-KL5 carbapenem-resistant *Klebsiella pneumoniae* with a *bla*_KPC-2_ and *bla*_NDM-1_ co-carrying hypertransmissible IncM1 plasmid

**DOI:** 10.1038/s42003-024-06398-w

**Published:** 2024-06-06

**Authors:** Hongmao Liu, Yating Xiang, Mengyuan Xiong, Xiao Xiao, Junying Zhou, Hongpan Tian, Qingsong Chen, Yirong Li

**Affiliations:** 1 Department of Laboratory Medicine, Zhongnan Hospital of Wuhan University, Wuhan, China; 2 Hubei Engineering Center for Infectious Disease Prevention, Control and Treatment, Wuhan, China; 3 Wuhan Research Center for Infectious Diseases and Tumors of the Chinese Academy of Medical Sciences, Wuhan, Hubei China

**Keywords:** Antimicrobial resistance, Bacterial infection

## Abstract

Infection caused by KPC and NDM carbapenemases co-producing *Klebsiella pneumoniae* (KPC_NDM_CRKP) poses serious public health concerns. Here, we elucidate the prevalence of a hypertransmissible lncM1 plasmid, pKPC_NDM, co-carrying *bla*_KPC-2_ and *bla*_NDM-1_ genes in sequence type 1049 K_locus 5 (ST1049-KL5) KPC_NDM_CRKP isolates. Genetic and clonal relatedness analyses using pulsed-field gel electrophoresis, single nucleotide polymorphism analysis and core genome multilocus sequence typing suggested clonal dissemination of ST1049-KL5 KPC_NDM_CRKP strains in our hospital. Whole genome sequencing identified an identical 76,517 bp- *bla*_KPC-2_ and *bla*_NDM-1_ genes co-carrying IncM1 plasmid pKPC_NDM and a pLVPK-like hypervirulent plasmid in all ST1049-KL5 KPC_NDM_CRKP isolates. pKPC_NDM shared 100% identity with a previously sequenced plasmid CRKP35_unnamed4, demonstrating high transferability in conjugation assay, with conjugation frequencies reaching 10^-4^ and 10^-5^ in *Escherichia coli* and *K. pneumoniae* recipients, respectively. It also maintained favorable stability and flexible compatibility, with retention rates exceeding 80% after 10 days of continuous passage, and could be compatible with pre-existing *bla*_KPC_- or *bla*_NDM_-carrying plasmids in recipient strains. This study summarizes the characteristics of KPC_NDM_CRKP outbreaks and highlights the importance of ongoing surveillance and infection control strategies to address the challenges posed by ST1049 *K. pneumoniae* strains.

## Abstract (web summary)

The study focuses on a *bla*_KPC-2_ and *bla*_NDM-1_ co-carrying hypertransmissible IncM1 plasmid in ST1049 CRKP strains, highlights the importance of ongoing surveillance and infection control strategies to address the challenges posed by ST1049 *K. pneumoniae*.

## Introduction

*Klebsiella pneumoniae* is a crucial opportunistic pathogen responsible for both community-acquired and nosocomial infections. Infections caused by *K. pneumoniae*, especially those associated with carbapenem resistance and hypervirulence, can result in high morbidity and mortality rates^1^. The primary mechanism underlying carbapenem resistance involves the production of carbapenemase, of which *K. pneumoniae* carbapenemase (KPC) and New Delhi metallo-β-lactamase (NDM) are the two most commonly encountered types. The hydrolytic activity of KPC can be inhibited using diazabicyclooctane inhibitors such as avibactam (AVI), relebactam, and the cyclic boronic acid pharmacophore inhibitor vaborbactam; by contrast, the presence of NDM negatively impacts the use of these inhibitors^2^.

The co-production of KPC and NDM carbapenemases has been extensively documented across various *Enterobacteriaceae* species, encompassing *Escherichia coli*^3^, *Klebsiella oxytoca*^4^, *Klebsiella michiganensis*^5^, *Citrobacter portucalensis*^6^, *Enterobacter cloacae*^7^, and *K. pneumoniae*^8^. These bacteria exhibit resistance to nearly all commonly employed β-lactam/β-lactamase inhibitor combinations and carbapenems. While alternative therapeutic options such as aminoglycosides, colistin, and tigecycline may offer efficacy, the potential risks of nephrotoxicity and increased mortality associated with these agents cannot be disregarded^9^.

Previous research has primarily concentrated on elucidating the emergence of KPC and NDM co-producing carbapenem-resistant *K. pneumoniae* (KPC_NDM_CRKP)^10,11^. However, a comprehensive exploration of the epidemiological characteristics surrounding their outbreaks is lacking. Furthermore, while the presence of *bla*_KPC-2_ and *bla*_NDM-1_ genes has conventionally been associated with separate plasmids^10–12^, research investigating the coexistence of these two carbapenemase genes within the same plasmid, along with their potential synergistic effects, remains limited. Recently, Hu et al. reported the co-production of these two carbapenemases in a sequence type (ST) 1049 carbapenem-resistant *K. pneumoniae* (CRKP) strain, CRKP35, at our hospital^13^. Despite this important finding, minimal attention has been directed towards this uncommon sequence type thus far. Herein, we identified a conjugative IncM1 plasmid, pKPC_NDM, co-carrying the *bla*_KPC-2_ and *bla*_NDM-1_ carbapenemase genes in four ST1049-KL5 *K. pneumoniae* isolates. This plasmid exhibited high intraspecies- and interspecies transferability and enhanced carbapenem resistance, thus representing a potent threat to the prevention of CRKP.

## Results

### Clinical characteristics of patients with *K. pneumoniae* infection

All patients included in this study exhibited severe underlying conditions, such as neurological disorders and liver cirrhosis, compounded by *K. pneumoniae* infections, including pneumonia, urinary tract infection, or septicopyemia. Antimicrobial treatments comprised β-lactam/β-lactamase inhibitor combinations, carbapenems, and last-resort options such as polymyxin B and tigecycline. Patients infected with ST1049 KPC_NDM_CRKP exhibited poorer outcomes, with two of four patients succumbing to multiple organ infections and septicemia. Conversely, patients with ST11 KPC_NDM_CRKP infection exhibited more favorable recoveries. Detailed clinical characteristics are presented in Table 1.

**Table 1:** Clinical characteristics of patients with *K. pneumoniae* infection

Patients	Isolates	Sequence types	Age/Gender	Wards	Underlying diseases	Specimen	Isolated time	Days in hospital	Empirical treatment	Discharge status
1	KP3594	ST1049	32/Male	Neurological rehabilitation	Severe pneumonia, neuromyelitis spectrum disease	Sputum	September, 2020	60	TZP, BPM, IPM, PB, CAZ	Unhealed
2	KP1527	ST1049	30/Male	ICU	Craniocerebral injury, hypostatic pneumonia	Sputum	January, 2021	7	TZP	Died
3	KP2094	ST1049	56/Male	Hepatobiliary surgery	Infectious pneumonia, liver cirrhosis	Blood	January, 2021	38	IPM, TIG, PB	Died
4	KP1078	ST1049	38/Male	Neurosurgery	Infectious pneumonia, cerebral hernia	Sputum	June, 2021	54	CSL, TIG, PB, CAZ/AVI	Unhealed
5	KP2136	ST1049	67/Male	ICU	Subarachnoid hemorrhage	Sputum	April, 2022	16	Unused	Improved
6	KP2316	ST1049	67/Female	Rheumatology and immunology department	Infectious pneumonia	Bronchoalveolar lavage fluid	April, 2022	92	CAZ	Improved
7	KP4007	ST1049	63/Female	ICU	Acute suppurative cholangitis, septicopyemia	Drainage fluid	May, 2022	23	CSL, MXF	Improved
8	KP2963	ST11	83/Male	Neurological rehabilitation	Urinary tract infection, cerebral infarction	Urine	September, 2022	86	AMC	Improved
9	KP2310	ST11	24/Female	Nephrology	Acute renal failure, infectious pneumonia	Urine	August, 2020	25	IPM, CAZ/AVI	Improved
10	KP3813	ST11	73/Female	ICU	Severe pneumonia, septicopyemia	Urine	September, 2020	267	IPM, CSL, PB, CAZ/AVI	Died
11	KP2805	ST11	30/Female	Hepatopancreatobiliary surgery	Duodenal stump leakage, pneumono-abdominal infection	Drainage fluid	March, 2023	86	CSL, IMP, PB, COL, TZP, TIG, CAZ/AVI	Improved
12	KP2258	ST11	68/Female	Neurological rehabilitation	Urinary tract infection	Urine	April, 2023	83	MXF, TZP, AMK	Unhealed

*ICU* intensive care unit, *TZP* piperacillin-tazobactam, *BPM* biapenem, *IPM* imipenem, *PB* polymyxin B, *CAZ* ceftazidime, *TIG* tigecycline, *CSL* cefoperazone/sulbactam, *CAZ/AVI* ceftazidime/avibactam, *AMC* amoxicillin/clavulanate, *COL* colistin, *MXF* moxifloxacin, *AMK* amikacin.

### Clonal and genetic relatedness of *K. pneumoniae* isolates

**Fig. 1:**
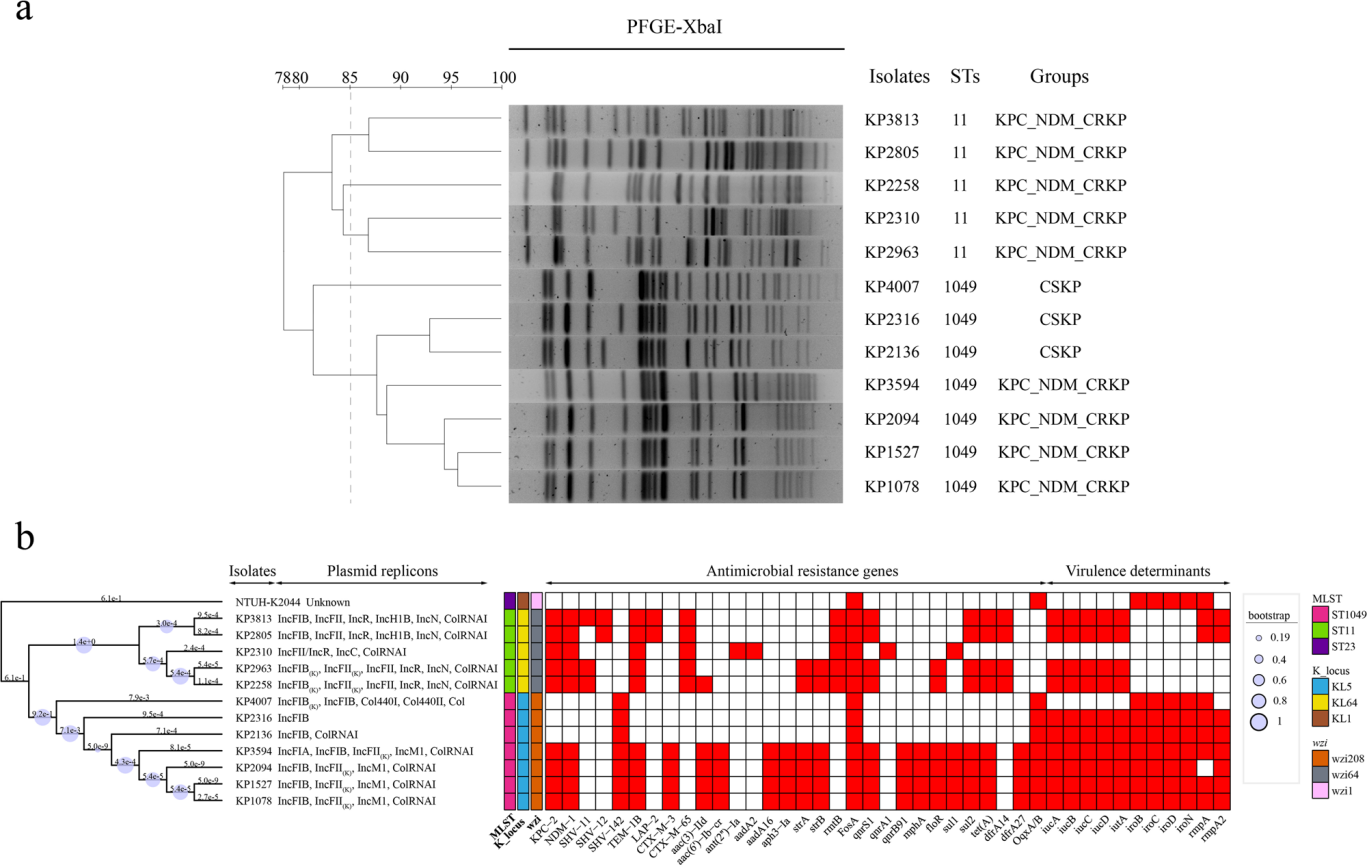
Clonal and genetic relatedness of clinical *K. pneumoniae* strains. **a** A dendrogram of PFGE profiles of *K. pneumoniae* strains. **b** Core genome single nucleotide polymorphism (cgSNP) analysis and distribution of plasmid replicons, antimicrobial resistance, and virulence genes.

Pulsed-field gel electrophoresis (PFGE) analysis indicated high clonal relatedness (with over 85% similarity) among all ST1049 *K. pneumoniae* isolates, except for KP4007 (Fig. 1a). Isolates KP1527, KP2094 and KP1078 exhibited identical electrophoretic bands, differing by only two bands from the initial isolate KP3594, suggesting clonal dissemination of ST1049 *K. pneumoniae* within our hospital. The construction of a core genome single nucleotide polymorphism (cgSNP)-based phylogenetic tree further corroborated these findings, revealing close genetic relationships (genetic distance < 0.001) among ST1049 KPC_NDM_CRKP strains, with minimal SNP variations (Fig. 1b). In contrast, while the ST11 KPC_NDM_CRKP group shared genetic homology in phylogenetic profiles, they displayed clonal heterogeneity in PFGE patterns.

### Comparative genomic analysis of the plasmids in ST1049 KPC_NDM_CRKP

**Fig. 2:**
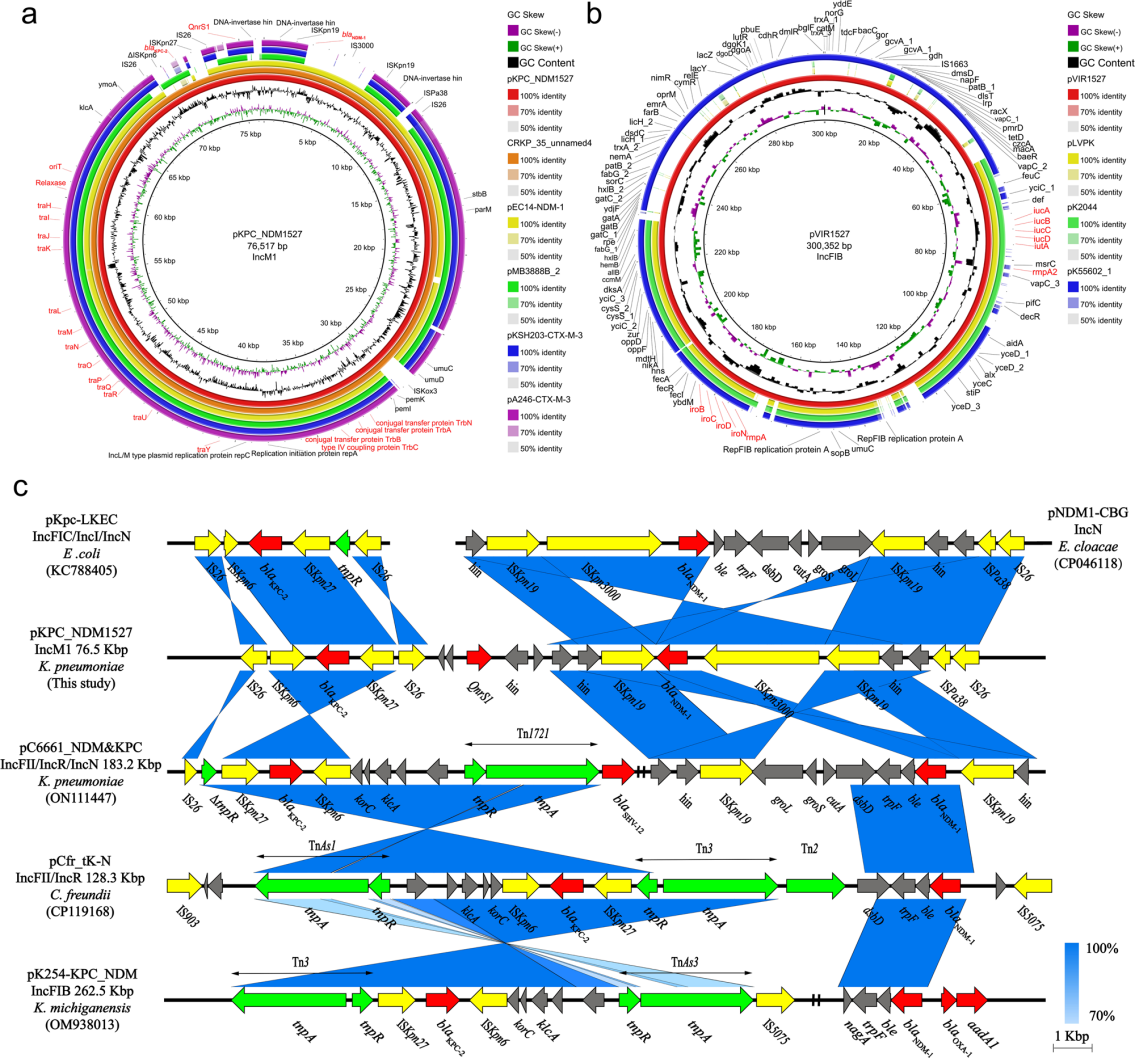
Comparative genomic analysis of *bla*_KPC-2_- and *bla*_NDM-1_-co-harboring plasmid (pKPC_NDM1527) and virulent plasmid (pVIR1527) in ST1049 KPC_NDM_CRKP strain KP1527 with publicly available high homologous plasmids. **a** Comparative genomic analysis of pKPC_NDM1527. Sequences from the inside toward the outside represent the following: pKPC_NDM1527, CRKP_35_unnamed4 (CP107356), pEC14-NDM-1 (CP060926), pMB3888B_2 (CP103692), pKSH203-CTX-M-3 (CP034325.) and pA246-CTX-M-3 (MT265678). Conjugative modules and antibiotic-resistance genes are indicated in red. **b** Comparative genomic analysis of pVIR1527 with hypervirulent reference plasmids pLVPK (AY378100), pK2044 (CP026012) and pK55602_1 (CP042975). Virulence genes were indicated in red. **c** Linear comparison of the *bla*_KPC-2_- and *bla*_NDM-1_-co-harboring regions of pKPC_NDM1527 with publicly available pKPC_NDM plasmids obtained from the NCBI Nucleotide database. Genes with different functions are indicated in different colors: red, antibiotic resistance genes; yellow, insertion sequence elements; green, transposons.

Illumina sequencing and whole genome sequencing (WGS) revealed high genomic diversities in the antimicrobial resistance and virulence gene profiles among KPC_NDM_CRKP isolates (Fig. 1b). Specifically, *bla*_KPC-2_ was identified in an IncFII/IncR multidrug-resistant plasmid, whereas *bla*_NDM-1_ resided in another IncN or IncC plasmid in ST11 KPC_NDM_CRKP isolates. By contrast, WGS analysis revealed that all ST1049 KPC_NDM_CRKP isolates harbored a 76,517 bp-IncM1 plasmid (pKPC_NDM) co-carrying the *bla*_KPC-2_, *bla*_NDM-1_ and *qnrS1* genes (Fig. 2a); an IncFII_(K)_ multidrug-resistant plasmid carrying a series of antimicrobial resistance genes and broad-spectrum β-lactamase genes; and an IncFIB plasmid-encoding virulence factors (Fig. 2b). The virulent plasmid pVIR1527 encompassed all plasmid-encoding virulence genes found in officially recognized hypervirulent plasmids pLVPK and pK2044 (with 55% coverage and 99% identity), and exhibited high similarity (81% coverage and 99.99% identity) with plasmid pK55602_1 obtained from *K. pneumoniae* strain KPN55602 (Fig. 2b). Notably, plasmid pKPC_NDM was identical to the previously deposited plasmid CRKP_35_unnamed4 from our hospital. It also exhibited a high similarity with the *bla*_NDM-1_-carrying IncM2 plasmid pEC14-NDM-1 (Genbank accession number CP060926) obtained from *E. coli* strain EC14 in Zhejiang province, with 93% coverage and 93.37% identity (Fig. 2a). The other IncM1 plasmids carried *bla*_KPC-2_ or *bla*_CTX-M-3_ genes. Moreover, pKPC_NDM1527 contained a set of mobile genetic elements associated with conjugation, such as the origin site of DNA transfer (*oriT*), relaxase, bacterial type IV secretion system (T4SS)-encoding gene clusters (*tra* genes), and type IV coupling proteins (T4CP)-encoding gene clusters (*trb* genes). A linear comparison of pKPC_NDM with plasmids from the NCBI GenBank database highlighted the conservation of the *bla*_KPC-2_ region and diversity in the *bla*_NDM-1_ region (Fig. 2c). Specifically, the *bla*_KPC-2_ bearing region in pKPC_NDM resembled the previously reported NTE_KPC_-Id structure identified in pKpc-LKEC (Genbank accession number KC788405), whereas the *bla*_NDM-1_ region shared the highest similarity with that of IncN plasmid pNDM1-CBG (Genbank accession number CP046118).

### Characterization of transconjugants/transformants and validation of pKPC_NDM transferability

**Fig. 3:**
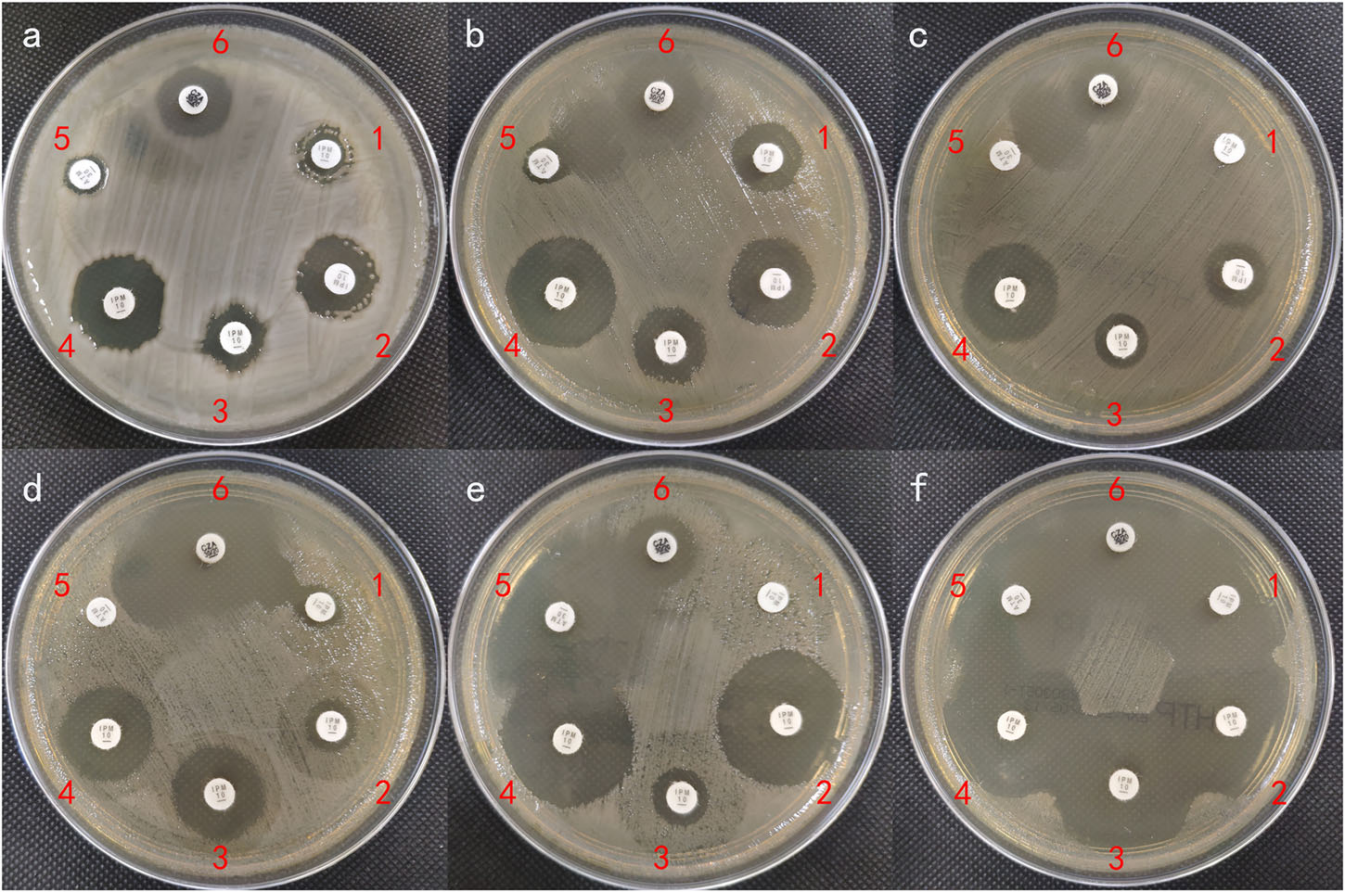
Phenotypic carbapenemase confirmation using the APB/EDTA enhancement method. **a** KP1527. **b** EC600::pKPC_NDM1527. **c** Top10::pMD19-KPC_NDM. **d** Top10::pMD19-*bla*_KPC-2_. **e** Top10::pMD19-*bla*_NDM-1_. **f** Top10::pUC19. Numbers in the clockwise direction represent the following: 1, IPM (imipenem); 2, IPM + EDTA; 3, IPM + APB (3-aminophenylboronic acid); 4, IPM + EDTA + APB; 5, ATM (aztreonam); 6, CAZ/AVI (ceftazidime/avibactam).

The successful transfer of pKPC_NDM from donor ST1049 KPC_NDM_CRKP strains to recipient *E. coli* EC600 demonstrated a higher transmission frequency compared to the *bla*_KPC-2_- or *bla*_NDM-1_-carrying plasmids of ST11 KPC_NDM_CRKP (Supplementary Data 2). pKPC_NDM could be further transferred from ST1049 KPC_NDM_CRKP to carbapenem-sensitive *K. pneumoniae* (CSKP) control *K. pneumoniae* ATCC 700603, with a conjugation frequency of (8.67 ± 1.14) × 10^-4^, and from the donor *E. coli* EC600::pKPC_NDM to ST1049 CSKP recipients. Detailed characteristics of transconjugants were listed in Supplementary Data 2. Carbapenemase confirmation assays validated the co-production of KPC and NDM carbapenemases in both the wild strain and pKPC_NDM-carrying transconjugants/transformants (Fig. 3). Notably, the presence of ghost zones between aztreonam (ATM) and ceftazidime/avibactam (CAZ/AVI) disks suggested a synergistic interaction against pKPC_NDM-carrying strains (Figs. 3a–c).

A comparative analysis of the *oriT* derived from pKPC_NDM (designated as *oriT*_KN_) against *oriT* database (oriTDB) in oriTFinder program revealed its highest similarity (H-value = 0.92) with *oriT*_pCTXM360 (oriTDB accession number 100105) from the broad-host-range conjugative IncM2 plasmid pCTXM-360 (NCBI accession number NC_011641)^14^. Further comparison with 90 plasmids retrieved from the NCBI database, all sharing identical *oriT*_KN_ sites, indicated a strong correlation between *oriT*_KN_ and IncM1 conjugative plasmids (Supplementary Data 3), underlining its crucial role in the transmission of IncM1 plasmids. Additionally, the insertion of *oriT*_KN_ facilitated the spontaneous conjugation of pUCP24 from *E. coli* S17 to *E. coli* EC600 and *K. pneumoniae* ATCC 700603, with frequencies of (1.27 ± 0.25) × 10^-5^ and (1.93 ± 0.52) × 10^-5^, respectively (Supplementary Data 2).

### Stability, incompatibility, and carbapenem resistance maintenance in pKPC_NDM recipients

Considering the hypertransmissibility of pKPC_NDM, which facilitates its mobilization across hosts of different species, we conducted plasmid stability and fitness assays. The results demonstrated that pKPC_NDM exhibited robust stability when introduced into ST1049 CSKP recipients and *E. coli* EC600 through conjugation, with retention rates exceeding 80% after 10 consecutive days of passage (Supplementary Fig. S1a). Importantly, this stability did not compromise fitness, as evidenced by comparable growth curves between transconjugants and their wild strain counterparts (Supplementary Fig. S1b). However, the carbapenem resistance maintenance results showed that 3.9% (5/128) of *E. coli* recipients and 2.3% (3/128) of *K. pneumoniae* recipients lost carbapenem resistance during passage, and all these strains lost the entire pKPC_NDM plasmid (Supplementary Fig. S2). Intriguingly, one particular transconjugant maintained reduced carbapenem resistance but lost CAZ/AVI resistance (Supplementary Fig. S2a), sequencing results identified the loss of the *bla*_NDM-1_ gene region (designated as pKPC_NDMΔNDM, available at NCBI accession number CP149795). Further experimentation involved successfully knocking out the *bla*_KPC-2_ gene (designated as pKPC_NDMΔKPC) using the CRISPR-Cas9 method, but attempts to knock out the *bla*_NDM-1_ gene resulted in the loss of the entire pKPC_NDM plasmid in the host strain. Moreover, plasmids with KPC- or NDM-region deletions showed no substantial difference in conjugation efficiency compared to pKPC_NDM (Supplementary Data 2). In the incompatibility assay, pKPC_NDM was found to be compatible with the pre-existing IncFII/IncR *bla*_KPC-2_ and IncN or IncC *bla*_NDM-1_ plasmids in KPC- or NDM-producing CRKP strains, with all transformant colonies retaining pKPC_NDM along with the *bla*_KPC-2_- or *bla*_NDM-1_-carrying plasmids after culture on antibiotic-free plates.

### Antimicrobial susceptibility testing (AST) results

KPC_NDM_CRKP strains exhibited resistance to all clinically used β-lactams, including cephalosporins (cefepime and ceftazidime), monobactam (ATM), β-lactam/β-lactamase inhibitor combinations (cefoperazone/sulbactam, piperacillin/tazobactam and CAZ/AVI), and carbapenems (imipenem, meropenem and biapenem). However, they remained susceptible to last-resort antibiotics such as polymyxin B and tigecycline and displayed varying resistance patterns to aminoglycosides (gentamicin, tobramycin, and amikacin), quinolones (ciprofloxacin) and trimethoprim-sulfamethoxazole (Table 2). Although the potency of CAZ/AVI was limited, combination therapies of AVI with ATM or mecillinam (MEC) showed substantial improvements against KPC_NDM_CRKP strains. Furthermore, a two-fold decrease in carbapenem MICs was observed when the *bla*_KPC_ or *bla*_NDM_-bearing regions were deleted.

**Table 2:** Antimicrobial susceptibility testing results of clinical *K. pneumoniae* isolates and transconjugants/transformants (mg/L)

Strains	Descriptions	β-lactams				β-lactam/β-lactamase inhibitor combinations					Carbapenems			Aminoglycosides			Other antibiotics			
		MEC	CAZ	FEP	ATM	CSL	TZP	MEC/AVI	CAZ/AVI	ATM/AVI	IPM	MEM	BPM	GEN	TOB	AMK	CIP	SXT	TIG	PB
KP3594^a^	Clinical ST1049 KPC_NDM_CRKP isolate	>256	>256	64	>256	>256	>256	1	>256	0.125	128	128	64	32	4	1	32	>608/32	8	1
KP1527^a^	Clinical ST1049 KPC_NDM_CRKP isolate	>256	>256	64	>256	>256	>256	1	>256	≤0.125	128	256	128	32	4	1	8	>608/32	2	2
KP2094^a^	Clinical ST1049 KPC_NDM_CRKP isolate	>256	>256	64	>256	>256	>256	1	>256	0.125	128	256	128	32	4	2	16	>608/32	4	1
KP1078^a^	Clinical ST1049 KPC_NDM_CRKP isolate	>256	>256	64	>256	>256	>256	1	>256	≤0.125	128	256	128	32	4	2	8	>608/32	2	1
KP2136^b^	Clinical ST1049 CSKP isolate	4	≤0.125	≤0.125	≤0.125	0.25	1	0.125	≤0.125	≤0.125	0.5	0.5	0.5	0.5	0.25	2	≤0.125	≤19/1	1	1
KP2316^b^	Clinical ST1049 CSKP isolate	4	≤0.125	≤0.125	≤0.125	0.25	1	0.125	≤0.125	≤0.125	0.5	≤0.125	0.5	0.5	0.25	2	≤0.125	≤19/1	2	2
KP4007^b^	Clinical ST1049 CSKP isolate	4	1	≤0.125	≤0.125	≤0.125	2	≤0.125	≤0.125	≤0.125	0.5	≤0.125	0.25	0.5	0.5	1	≤0.125	≤19/1	1	1
KP2963^a^	Clinical ST11 KPC_NDM_CRKP isolate	>256	>256	>256	>256	>256	>256	16	>256	0.5	256	>256	>256	>256	>256	>256	128	>608/32	8	1
KP2310^a^	Clinical ST11 KPC_NDM_CRKP isolate	>256	>256	>256	>256	>256	>256	4	>256	0.5	256	>256	>256	>256	>256	>256	256	≤19/1	1	1
KP3813^a^	Clinical ST11 KPC_NDM_CRKP isolate	>256	>256	>256	>256	>256	>256	4	>256	1	256	>256	>256	>256	>256	>256	256	>608/32	8	1
KP2805^a^	Clinical ST11 KPC_NDM_CRKP isolate	>256	>256	>256	>256	>256	>256	2	>256	0.5	256	>256	256	>256	>256	>256	256	>608/32	8	>128
KP2258^a^	Clinical ST11 KPC_NDM_CRKP isolate	>256	>256	>256	>256	>256	>256	4	>256	0.25	256	>256	>256	>256	>256	>256	64	>608/32	4	2
ATCC 25922	*E. coli* quality control strain	0.25	1	1	≤0.125	0.5	0.5	≤0.125	≤0.25	≤0.125	0.25	0.5	≤0.125	≤0.06	1	2	≤0.06	≤19/1	0.25	2
ATCC 700603^b^	*K. pneumoniae* control and conjugation recipient	4	16	8	16	0.25	0.5	0.25	0.25	0.125	0.25	≤0.125	≤0.125	2	0.5	1	128	>608/32	2	2
ATCC 700603::pKPC_NDM	*K. pneumoniae* transconjugant with pKPC_NDM	>256	>256	32	>256	>256	>256	1	>256	0.125	64	128	64	1	0.5	1	128	>608/32	2	2
KP2136::pKPC_NDM	*K. pneumoniae* transconjugant with pKPC_NDM	>256	>256	16	>256	>256	>256	1	>256	≤0.125	128	64	64	0.5	0.25	2	≤0.125	≤19/1	1	1
KP4007::pKPC_NDM	*K. pneumoniae* transconjugant with pKPC_NDM	>256	>256	32	>256	>256	>256	2	>256	≤0.125	64	64	64	0.5	0.5	0.5	≤0.125	≤19/1	1	1
KP2316::pKPC_NDM^a^	*K. pneumoniae* transconjugant with pKPC_NDM	>256	>256	16	>256	>256	>256	1	>256	≤0.125	128	64	128	0.5	0.25	2	≤0.125	≤19/1	2	2
KP2316::pKPC_NDMΔKPC^a^	*K. pneumoniae* transconjugant with *bla*_KPC-2_ deletion	2	>256	8	≤0.125	>256	>256	1	>256	≤0.125	64	16	64	0.5	0.25	2	≤0.125	≤19/1	2	2
KP2316::pKPC_NDMΔNDM^a^	*K. pneumoniae* transconjugant with *bla*_NDM-1_ deletion	>256	8	4	>256	>256	>256	0.25	0.25	≤0.125	32	32	64	0.5	0.25	2	≤0.125	≤19/1	2	2
EC600::pKPC_NDM^a^	*E. coli* transconjugant with pKPC_NDM	>256	>256	16	>256	>256	>256	1	>256	0.125	32	16	16	0.5	0.25	0.5	≤0.125	≤19/1	0.25	0.5
EC600::pKPC_NDMΔKPC	*E. coli* transconjugant with *bla*_KPC-2_ deletion	2	>256	16	≤0.125	>256	>256	1	>256	≤0.125	16	8	4	0.5	0.25	0.5	≤0.125	≤19/1	0.25	0.25
EC600::pKPC_NDMΔNDM	*E. coli* transconjugant with *bla*_NDM-1_ deletion	>256	16	8	>256	>256	>256	0.25	0.25	≤0.125	16	8	8	0.5	0.25	0.5	≤0.125	≤19/1	0.25	0.5
EC600::pKPC2963	*E. coli* transconjugant with pKPC2963	>256	>256	16	>256	256	>256	0.25	>256	0.125	32	16	16	>256	>256	>256	0.25	≤19/1	0.25	0.5
EC600::pNDM2963	*E. coli* transconjugant with pNDM2963	4	>256	16	0.5	256	>256	1	>256	0.125	16	16	4	1	1	2	4	≤19/1	0.25	0.5
EC600::pKPC2310	*E. coli* transconjugant with pKPC2310	>256	>256	16	>256	256	256	0.25	0.5	0.125	8	8	8	>256	>256	>256	0.25	≤19/1	0.25	0.5
EC600::pNDM2310	*E. coli* transconjugant with pNDM2310	4	>256	16	0.25	256	256	1	>256	0.125	8	8	2	8	8	2	2	≤19/1	0.25	0.5
EC600::pNDM3813	*E. coli* transconjugant with pNDM3813	4	>256	16	0.25	256	>256	1	>256	0.125	16	16	8	1	1	2	4	≤19/1	0.25	0.5
EC600::pKPC2805	*E. coli* transconjugant with pKPC2805	>256	>256	16	>256	>256	>256	1	1	0.25	16	16	8	>256	>256	>256	0.5	≤19/1	0.25	0.5
EC600::pNDM2805	*E. coli* transconjugant with pNDM2805	4	>256	16	0.5	256	256	0.5	>256	0.125	8	8	8	2	1	2	2	≤19/1	0.25	0.5
EC600::pKPC2258	*E. coli* transconjugant with pKPC2258	>256	>256	16	>256	256	256	1	0.5	0.25	8	8	8	>256	>256	>256	0.25	≤19/1	0.25	0.5
EC600^b^	*E. coli* conjugation recipient	0.25	1	≤0.125	0.5	0.25	0.25	≤0.125	≤0.125	≤0.125	0.25	≤0.125	≤0.125	0.5	0.25	0.5	≤0.125	≤19/1	0.25	0.5
Top10::pMD19-*bla*_KPC-2_	*E. coli* transformant with *bla*_KPC-2_ gene	>256	>256	32	>256	>256	>256	1	1	0.125	32	32	32	1	1	2	≤0.125	≤19/1	0.25	0.5
Top10::pMD19-*bla*_NDM-1_	*E. coli* transformant with *bla*_NDM-1_ gene	2	>256	16	1	256	256	1	>256	≤0.125	4	4	2	1	1	2	≤0.125	≤19/1	0.25	0.5
Top10::pMD19-KPC_NDM	*E. coli* transformant with *bla*_KPC-2_ and *bla*_NDM-1_ genes	>256	>256	128	>256	>256	>256	1	>256	0.125	64	64	64	1	1	2	≤0.125	≤19/1	0.25	0.5
Top10::pUC19	*E. coli* transformant with the empty vector	16	1	0.5	1	2	2	≤0.125	0.25	≤0.125	≤0.125	≤0.125	≤0.125	1	1	2	≤0.125	≤19/1	0.25	0.5
Top10	*E. coli* transformation recipient	0.25	0.5	0.25	≤0.125	0.125	0.25	≤0.125	≤0.125	≤0.125	0.25	≤0.125	≤0.125	1	1	2	≤0.125	≤19/1	0.25	0.5

*CAZ* ceftazidime, *CAZ/AVI* ceftazidime/avibactam, *ATM* aztreonam, *ATM/AVI* aztreonam/avibactam, *MEC* mecillinam, *MEC/AVI* mecillinam/avibactam, *IPM* imipenem, *MEM* meropenem, *BPM* biapenem, *FEP* cefepime, *CSL* cefoperazone/sulbactam, *TZP* piperacillin/tazobactam, *CIP* ciprofloxacin, *GEN* gentamicin, *TOB* tobramycin, *AMK* amikacin, *SXT* trimethoprim-sulfamethoxazole, *TIG* tigecycline, *PB* polymyxin B, *KPC_NDM_CRKP* KPC and NDM co-producing carbapenem-resistant *K. pneumoniae*, *CSKP* carbapenem-susceptible *K. pneumoniae*.

^a^Donor strains in conjugation experiment.

^b^Recipient strains in conjugation experiment. Detailed characteristics for transconjugants are available in Supplementary Data 2.

### Pathogenicity assessment and time-kill assay results

**Fig. 4:**
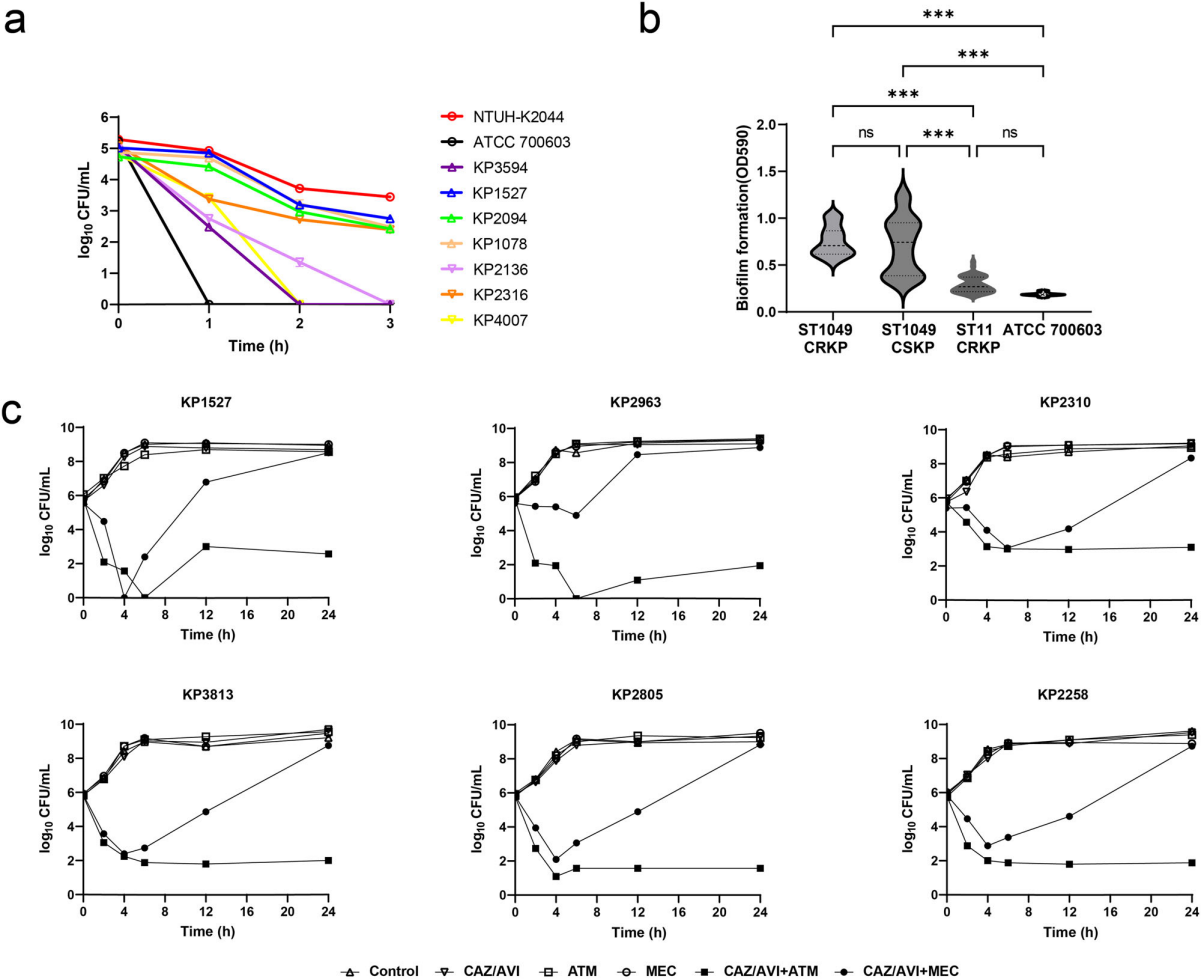
Pathogenicity characterization and time-kill curves of clinical *K. pneumoniae* strains. **a** Serum resistance assay of ST1049 *K. pneumoniae* strains. **b** Biofilm formation of clinical *K. pneumoniae* strains. **c** Time-kill curves of ceftazidime/avibactam (CAZ/AVI) monotherapy and combined treatments with aztreonam (ATM) or mecillinam (MEC) against KPC_NDM_CRKP strains. *** indicates *p*-value < 0.001 by one-way ANOVA.

Most KPC_NDM_CRKP isolates demonstrated high sensitivity to human serum (Fig. 4a). However, the overall biofilm production of ST1049 *K. pneumoniae* was significantly higher than that of the ST11 *K. pneumoniae* strains (Fig. 4b, *p* < 0.001). Although there was no significant difference in the biofilm formation abilities of the ST1049 KPC_NDM_CRKP and CSKP groups, the former exhibited enhanced resistance to serum killing (*p* = 0.04). Time-kill assays revealed limited efficacy of CAZ/AVI, ATM, or MEC monotherapy in vitro against KPC_NDM_CRKP strains, whereas combination therapies demonstrated synergistic effects (Fig. 4c).

### Core genome multi-locus sequence typing (cgMLST) analysis of ST1049 *K. pneumoniae* and KPC_NDM_CRKP strains

**Fig. 5:**
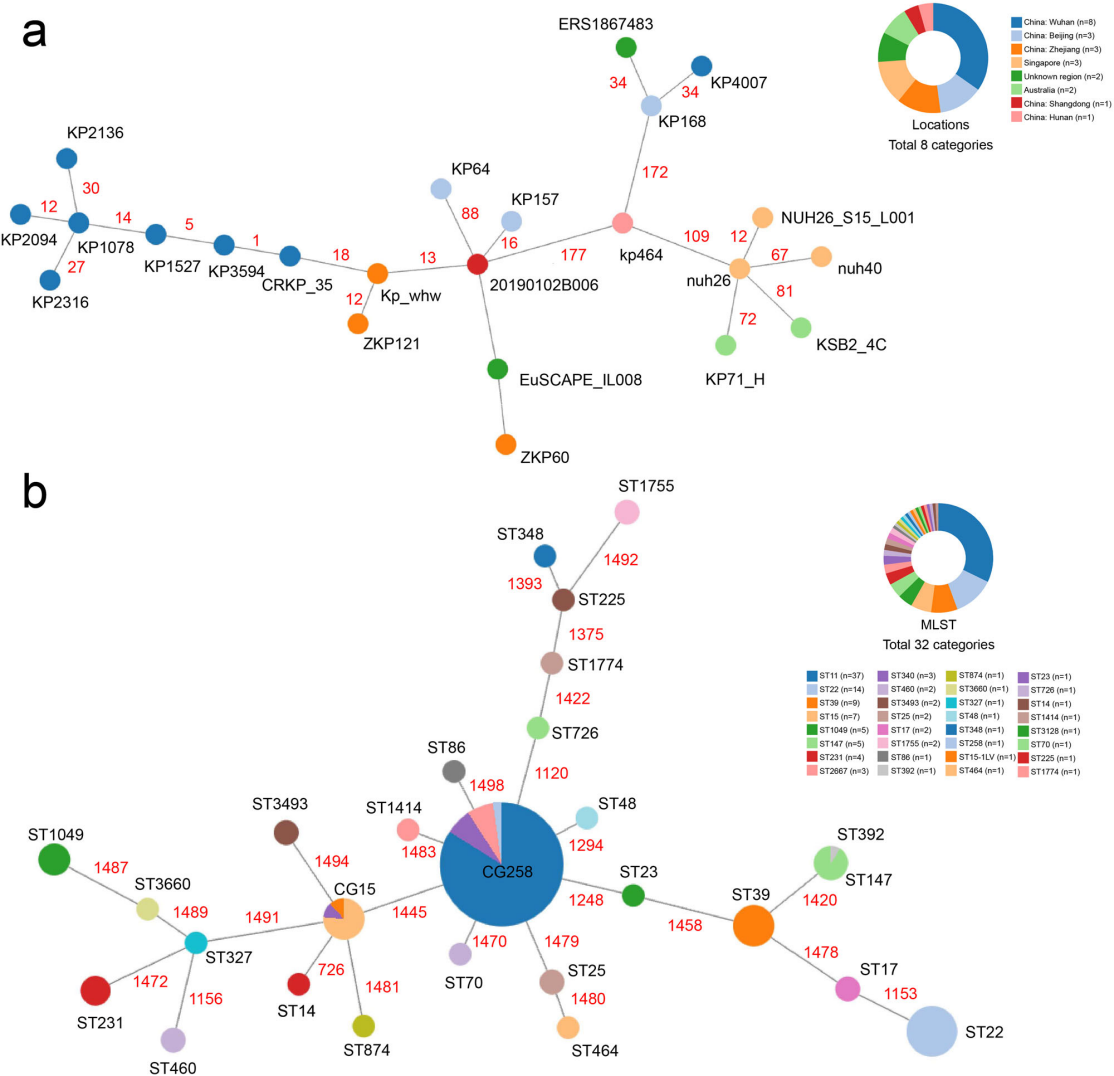
Core genome multi-locus sequence typing (cgMLST) analysis of *K. pneumoniae* strains. **a** Distribution of ST1049 *K. pneumoniae* strains differentiated by locations. **b** Distribution of KPC_NDM_CKKP strains differentiated by MLST sequence types. The minimum-spanning tree was constructed with the collapsed distance set at 395. Absolute distance is indicated in red font.

A total of 138 *K. pneumoniae* genomes, comprising 23 ST1049 strains and 115 KPC_NDM_CRKP strains of other sequence types, were included for cgMLST analysis. The minimum-spanning tree identified that the ST1049 epidemic was regionally confined, primarily in China, with remarkable diversity among the KPC_NDM_CRKP strains (Fig. 5, Supplementary Data 4 and  5). The prevalence of KPC_NDM_CRKP comprised several successful clones (Fig. 5b), indicating both genomic and geographic heterogeneity. Intriguingly, the outbreak of ST1049 KPC_NDM_CRKP was initially and exclusively observed in our hospital.

## Discussion

In this study, we identified a hypertransmissible IncM1 plasmid co-harboring *bla*_KPC-2_ and *bla*_NDM-1_ genes in ST1049-KL5 KPC_NDM_CRKP strains. Conjugation assays revealed efficient transferability of pKPC_NDM between *K. pneumoniae* and *E. coli* hosts without compromising fitness. With a size of 76,517 bp, pKPC_NDM falls within the size range of pKPC and pNDM plasmids found in ST11 KPC_NDM_CRKP isolates, making it the smallest publicly available *bla*_KPC-2_- and *bla*_NDM-1_-co-carrying conjugative plasmid, to the best of our knowledge (Fig. 2c). It also confers enhanced carbapenem resistance and transferability compared with pKPC and pNDM in *E. coli* recipients (Table 2, Supplementary Data 2). Comparative analysis and cloning of pKPC_NDM-derived *oriT* further suggested its close correlation with IncM1 conjugative plasmids (Supplementary Data 3) and contribution to the spontaneous conjugation of pUCP24 to *E. coli* and *K. pneumoniae* hosts. According to the conjugative mechanism^15,16^, a self-transmissible plasmid typically contains a complete set of conjugative elements, including *oriT*, relaxase, functional T4SS, and T4CP. The pKPC_NDM plasmid identified in this study contains all essential conjugative modules for conjugation (Fig. 2a), while the unsuccessful transconjugants (pKPC3813 and pNDM2258) in the conjugation assay lacked any of the essential elements except for the *oriT* sites (Supplementary Data 2). Taking into account all this evidence, we speculated that the favorable size, functional *oriT* site, and complete conjugal transfer elements contribute to the high transferability of pKPC_NDM. Furthermore, incompatibility assay demonstrated that pKPC_NDM is compatible with the pre-existing pKPC and pNDM plasmids in the CRKP strains of different sequence types. The high transmission ability, flexible compatibility, and stable maintenance in a broad range of bacterial hosts further facilitate the widespread dissemination of pKPC_NDM plasmids in *K. pneumoniae* and other *Enterobacteriaceae* strains, exacerbating the challenge posed by CRKP epidemics.

Transposition events mediated by non-Tn*4401* elements have been implicated in the rapid spread of the *bla*_KPC_ gene in *Enterobacteriaceae* strains in China^17^. In our study, we observed an IS*26*-mediated *bla*_KPC-2_ transporting structure in pKPC_NDM, resembling the NTE_KPC_-Id-like translocatable unit found in pKPC-LKEC and pKPC-CR-HvKP4_SH9 (IS*26*-ΔTn*3*-IS*Kpn8*-*bla*_KPC-2_-ΔIS*Kpn6*-*korC*-orf-IS*26*)^17,18^. Given the high similarity to plasmid pEC14-NDM-1, it is reasonable to speculate that the translocation of the IS*26*-mediated *bla*_KPC-2_-bearing composite transposon played an important role in transferring *bla*_KPC-2_ to a pEC14-NDM-1-like IncM plasmid. Unlike *bla*_KPC-2_, which is typically associated with IncFII plasmids and clonal group (CG) 258^19^, pKPC_NDM plasmids exhibit greater transmission flexibility, without a preference for particular host species or sequence types (Fig. 2c and Supplementary Data 2). A previous study suggested that KPC_NDM_CRKP may evolve from KPC-producing *K. pneumoniae* by acquiring *bla*_NDM_-carrying conjugative plasmids from a broad range of hosts^10^. However, our findings suggest that *bla*_NDM_-derived plasmids also have the opportunity to evolve into hybrid pKPC_NDM plasmids by acquiring the *bla*_KPC-2_-encoding region, posing serious clinical challenges.

To ascertain the clonal and genetic relatedness of *K. pneumoniae* strains, we employed PFGE, cgSNP, and cgMLST analyses. These analyses suggest that the transmission of ST1049 KPC_NDM_CRKP was likely a clonal event, originating from a single common ancestor, as evidenced by minimal SNP variants and identical PFGE patterns. CgMLST results further supported this, revealing a geographical concentration of ST1049 strains and high genetic similarity among those found in our hospital. This sequence type, first reported in the Zhejiang province of China^20^, has not received much attention thus far. However, our findings highlight the potential of ST1049 strains to harbor both hypervirulent and multidrug-resistant plasmids, emphasizing the need for ongoing surveillance and infection control measures. In contrast to the clonal dissemination of ST1049 KPC_NDM_CRKP strains, ST11 KPC_NDM_CRKP isolates appeared sporadically. Considering all publicly available KPC_NDM_CRKP isolates, we observed genomic and phenotypical heterogeneity, with ST11 (a well-known member of CG258)^19^ being the most prevalent, followed by ST22 and ST39. The diversity and complexity of infections caused by KPC_NDM_CRKP complicate treatment strategies, making this pathogen more resistant to treatment.

Previous studies have recommended combining ATM with CAZ/AVI against KPC_NDM_CRKP isolates, exploiting the susceptibility of NDM to ATM and the inhibition of KPC by AVI^8^. Our study confirmed these findings^21^, observing synergistic effects of ATM and CAZ/AVI against ST1049 KPC_NDM_CRKP isolates and pKPC_NDM-carrying *E. coli* transconjugants or transformants, as supported by the AST and time-kill assays. Furthermore, mecillinam/avibactam (MEC/AVI) showed antimicrobial activity comparable to that of aztreonam/avibactam (ATM/AVI), superior to CAZ/AVI monotherapy. However, the bactericidal effect of the CAZ + MEC + AVI combination was time-dependent, consistent with previous findings^21^. MEC is a semisynthetic penicillin primarily used for uncomplicated urinary tract infections. Although the production of KPC and VIM carbapenemases may lead to unexpected MEC resistance^22^, it still retains excellent activity against OXA-48- and NDM-1-producing *Enterobacterales*^23,24^. These results suggest that MEC/AVI could be a viable alternative for treating KPC_NDM_CRKP infections.

Our study has some limitations. We demonstrated that *oriT*_KN_ contributes to the transferability of pKPC_NDM and its high correlation with IncM1 conjugative plasmids. However, the precise relationship remains to be fully explored. Despite the high biofilm formation capabilities and high carriage rate of plasmid-encoding virulence genes, ST1049 strains did not exhibit comparable hypervirulence in the *in vivo Galleria mellonella* larva post-infection assay (Supplementary Fig. S3). Given the limited sample size, it is challenging to determine whether inherent or chromosomal mechanisms underlie the observed virulence heterogeneity in ST1049 *K. pneumoniae* strains.

In conclusion, the emergence and clonal dissemination of ST1049-KL5 CRKP carrying the *bla*_KPC-2_- and *bla*_NDM-1_-co-harboring IncM1 plasmid pKPC_NDM pose formidable public health concerns. This study highlights the potential for ST1049 strains to serve as carriers of both hypervirulent and multidrug-resistant plasmids, emphasizing the importance of continuous surveillance and the implementation of effective infection control strategies.

## Methods

### Bacterial isolates and identification

Continuous surveillance of carbapenemase production and phenotypes was conducted as part of our clinical routine at Zhongnan Hospital of Wuhan University^13,25^. Phenotypic carbapenemase production was confirmed by synergy tests using imipenem disks supplemented with 600 μg of 3-aminophenylboronic acid (APB) or/and 730 μg of EDTA (APB/EDTA enhancement method)^26^. Carbapenemase genotypes were further verified using polymerase chain reaction (PCR), amplifying carbapenemase-encoding genes including *bla*_KPC_, *bla*_NDM_, *bla*_IMP_, and *bla*_OXA-48-like_^27^. Between September 2020 and April 2023, a total of nine KPC and NDM carbapenemases co-producing *K. pneumoniae* strains were retrospectively collected from our clinical laboratory and previous study (out of 140 CRKP strains)^25^, this set comprising four ST1049 KPC_NDM_CRKP and five ST11 KPC_NDM_CRKP strains. Additionally, three ST1049 CSKP strains collected concurrently served as control strains. Strain identification was conducted using matrix-assisted laser desorption/ionization time-of-flight mass spectrometry (MALDI-TOF MS) (VITEK MS, bioMérieux). Clonal relatedness was determined using multi-locus sequence typing (MLST) and PFGE as previously described^28^. Clinical information was retrospectively gathered from electronic medical records. This study received approval from the medical ethics committee of Zhongnan Hospital of Wuhan University (2021128K).

### Antimicrobial susceptibility testing

AST was initially conducted using the VITEK II system (BioMérieux, Marcy l’Etoile, France) and subsequently confirmed using the agar dilution and broth microdilution methods. Results were interpreted according to the guidelines provided by the Clinical and Laboratory Standards Institute (CLSI, 2022) and the European Committee on Antimicrobial Susceptibility Testing (EUCAST) (http://www.eucast.org/). A minimum inhibitory concentration (MIC) of >4 mg/L was considered to indicate resistance to ATM/AVI^29^. Additionally, the CLSI breakpoint (of 16 mg/L) applied for MEC was further used for MEC/AVI. *E. coli* ATCC 25922 and *K. pneumoniae* ATCC 700603 served as the quality control strains.

### Conjugation and fitness evaluation

The transferability of pKPC_NDM plasmids in ST1049 KPC_NDM_CRKP, as well as the plasmids carrying *bla*_KPC-2_ or *bla*_NDM-1_ (designated as pKPC and pNDM, respectively) in ST11 CRKP, was assessed using conjugation assays. Recipients included rifampicin-resistant *E. coli* EC600 and CSKP strain ATCC 700603 (with induced rifampicin resistance). Successful *E. coli* conjugants (EC600::pKPC_NDM) were subsequently employed as donors for a second round of conjugation. The recipient strains comprised ST1049 CSKP isolates (KP2136, KP2316 and KP4007), IncFII/IncR *bla*_KPC-2_ plasmid-harboring ST11 CRKP strains (KP1878 and KP1880) and IncN or IncC *bla*_NDM-1_ plasmid-harboring CRKP strains (KP169 and KP253) in our previous research^28^. Transconjugants were selected on MacConkey agar plates supplemented with the combined antibiotics listed in Supplementary Data 2. The presence of *bla*_KPC-2_, *bla*_NDM-1_, and plasmid-encoded antimicrobial determinants in the recipients was confirmed using relevant primers listed in Supplementary Table S1. The conjugation frequency was calculated as the number of transconjugants per donor^30^. The fitness cost was assessed by conducting a growth curve assay on pKPC_NDM-carrying transconjugants^31^, with bacterial growth monitored by measuring the OD_600_ values every hour for 24 h.

### Plasmid stability and incompatibility assays

Plasmid stability and incompatibility assays were conducted based on previously described methods, with minor modifications^10^. For the stability assay, pKPC_NDM*-*harboring *E. coli* and *K. pneumoniae* transconjugants were cultured at 37 °C and subjected to 10 days of serial passage at a 1:1000 dilution in antibiotic-free BHI broth. The plasmid retention rate was determined by calculating the ratio of clones grown on meropenem-supplemented plates (2 mg/L) to those grown on antibiotic-free plates. To assess the maintenance of carbapenem resistance, 128 randomly selected colonies on day 10 were streaked onto antibiotic-free plates and plates supplemented with 2 mg/L MEM, 16 mg/L ATM, and 4 mg/L CAZ/AVI, respectively. Subsequently, carbapenem resistance profiles of the descendants were evaluated using an AST assay, and genotypes were validated using whole plasmid sequencing and long-range PCR, with primers spanning the *bla*_KPC-2_- and *bla*_NDM-1_-bearing regions as listed in Supplementary Table S1. Regarding the incompatibility assay, overnight cultures of conjugants containing pKPC_NDM, pKPC or pNDM were diluted and plated on antibiotic-free BHI agar. The presence of pKPC_NDM, pKPC, and pNDM plasmids in 40 randomly selected colonies was confirmed using the same determinants as in the conjugation assay. Plasmids were considered incompatible if over 80% of the colonies lost either of the plasmids.

### Genome editing

The deletion of *bla*_KPC-2_ and *bla*_NDM-1_ genes was conducted using the CRISPR-Cas9 system as previously described^32,33^. The apramycin-resistant ColRNAI plasmid pCasCure-apr was constructed using the plasmids pSGKP-km and pCasKp-apr as templates^33^. Annealed 20-nucleotide (nt) spacer oligonucleotides were then inserted into BsaI-digested pCasCure-apr using Golden Gate assembly^34^. Due to concerns regarding the incompatibility of the replicon of pCasCure-apr with the pre-existing ColRNAI plasmids, impeding its transformation into ST1049 KPC_NDM_CRKP strains, the pKPC_NDM recipient KP2316::pKPC_NDM was employed as the target strain for genome editing. Loss of targeted carbapenemase genes or plasmids was assessed by amplifying and sequencing the corresponding gene regions using the primers listed in Supplementary Table S1.

### Cloning experiment

A 9749 bp fragment containing the coding sequences and promoters of the *bla*_KPC-2_ and *bla*_NDM-1_ genes was amplified from the pKPC_NDM plasmid of ST1049 KPC_NDM_CRKP using LA Hot STAR (Takara, China) and the primer pairs LA-NDM-F and LA-KPC-R, as listed in Supplementary Table S1. These target sequences were then inserted into a linearized pMD19 vector and transformed into *E. coli* Top10 through chemical transformation. Additionally, the *bla*_KPC-2_ and *bla*_NDM-1_ genes with their respective promoters were individually cloned into the same vector. *E. coli* Top10 with the pUC19 vector was used as a control. Transformants were selected from BHI agar plates supplemented with 100 mg/L ampicillin, and the resulting recombinant plasmids, namely pMD19-KPC_NDM, pMD19-*bla*_KPC-2_, and pMD19-*bla*_NDM-1_, were verified through PCR and Sanger sequencing.

### Plasmid transferability

The *oriT* derived from pKPC_NDM was amplified and subsequently inserted into the shuttle vector pUCP24. The recombinant plasmid was then transformed into *E. coli* S17, a donor strain harboring the chromosomally integrated conjugative plasmid RP4-2, which encodes essential elements for bacterial conjugation^35^. Conjugation experiments were then conducted to assess the mobility of the recombinant plasmid, using *K. pneumoniae* ATCC 700603 and *E. coli* EC600 as recipient strains. Plasmids with identical *oriT* sites to pKPC_NDM were obtained from the NCBI Nucleotide database. Essential elements for bacterial conjugation were predicted and compared against the oriTFinder (including oriTDB) and VRprofile programs^36,37^.

### Pathogenicity and time-kill assay

Serum resistance and biofilm formation assays were conducted to assess the pathogenicity of the collected *K. pneumoniae* isolates. Biofilm was detected as previously described^38^, ATCC 700603 was selected as a negative control. Each assay was performed in triplicate. To determine the serum bactericidal activity, viable counts were checked after incubation in human serum for 0, 60, 120, and 180 min at 37 °C, the hypervirulent *K. pneumoniae* strain NTUH-K2044 was employed as a positive control strain^39^. The synergy of CAZ/AVI with ATM and MEC was determined through in vitro time-kill assays, performed in MH broth at an initial bacterial inoculum of 10^6^ CFU/ml for various time intervals (0, 2, 4, 6, 12, and 24 h) at 37 °C^40^. Synergy was defined as a ≥2-log_10_ CFU/mL reduction between the combination therapy and monotherapy. CLSI breakpoints for ATM and MEC, as recommended for *Enterobacteriaceae*, were adopted to compare monotherapy or combination therapy with 16 mg/L CAZ/AVI at fixed concentrations of 8 mg/L (for ATM) and 16 mg/L (for MEC). *K. pneumoniae* strain KP1527 was selected as a representative for ST1049 KPC_NDM_CRKP. For the *Galleria mellonella* larvae infection model, the mid-log-phase culture of ST1049 *K. pneumoniae* isolates was adjusted to 0.5 McFarland in PBS and then diluted to a final density of 10^6^ CFU/mL. Each larva (10 per group) was injected with 10 μL of bacterial suspension and survival proportions were recorded every 12 h^41^.

### Whole genome sequencing and bioinformatic analyses

Genomic DNA was extracted from clinical *K. pneumoniae* isolates using a bacterial genomic DNA extraction kit (Aidlab, China). The general genomic characteristics were comprehensively assessed using Illumina sequencing for all clinical strains, whereas WGS via the Pacbio platform was used to elucidate the detailed genetic contexts of all KPC_NDM_CRKP strains. Sequencing services were provided by Personal Biotechnology Co., Ltd (Shanghai, China). Genome assembly was conducted using the SPAdes v3.15.5 and Canu v2.2^42^. Antimicrobial resistance genes, virulence genes, and plasmid replicon types were identified by aligning the assembled genomes against the ResFinder, VFDB, and PlasmidFinder databases in CGE services (http://www.genomicepidemiology.org/) and Kleborate v2.3.2. Phylogenetic analyses utilized cgSNP analyses performed with Snippy v4.6.0 (https://github.com/tseemann/snippy) and cgMLST, with the *K. pneumoniae* NTUH-K2044 genome as a reference^43^. All publicly available ST1049 *K. pneumoniae* and KPC_NDM_CRKP genomes retrieved from the PATRIC database (as of 2023-07-01) were included for cgMLST analysis^44^. Minimum-spanning trees based on 1654 core alleles of the *K. pneumoniae* genome were constructed using chewBBACA v3.0.0 and visualized using the online PHYLOViZ v2.0 program^45^. Clonal groups were defined as groups with only one allelic mismatch in their MLST profiles^46^. Comparative genomic analysis was performed using BLAST Ring Image Generator (BRIG) v0.95 and EasyFig v2.2.3. The virulence and carbapenem-resistant plasmids of *K. pneumoniae* KP1527 were selected as representatives for ST1049 KPC_NDM_CRKP.

### Statistical analysis

SPSS 23.0 and GraphPad Prism 9.3.1 were used for statistical analysis. Biofilm formation, serum killing assay, and time-kill results were compared using one-way ANOVA. ^*^p < 0.05, ^**^p < 0.01, and ^***^p < 0.001 are considered statistically significant.

### Reporting summary

Further information on research design is available in the Nature Portfolio Reporting Summary linked to this article.

### Supplementary information

Supplementary Information

Description of additional supplementary files

Supplementary Data 1

Supplementary Data 2

Supplementary Data 3

Supplementary Data 4

Supplementary Data 5

Reporting summary

## Data Availability

All data used in this study are presented in this published article and supplementary files. Genome sequencing data is publicly available in the NCBI GenBank database under BioProject accession number PRJNA1019652.
