# Supplementary material for: Prevalence of ST1049-KL5 carbapenem-resistant Klebsiella pneumoniae with a blaKPC-2 and blaNDM-1 co-carrying hypertransmissible IncM1 plasmid
Source: Commun Biol. 2024 Jun 6;7:695. doi: 10.1038/s42003-024-06398-w (PMC11156905; doi:10.1038/s42003-024-06398-w)
Supplement: Supplementary file 1 — Supplementary Information [file 42003_2024_6398_MOESM1_ESM.pdf]

Supplementary Table S1. Primers used in this study.

Primers	Primer sequence (5'-3')	Size (bp)	Annealing (°C)	Description
pro-KPC-F	AAGATCTACAACCACAGCATTCCG	1,318	60	Amplifying the *bla*$_{KPC-2}$ gene with original promoter
pro-KPC-R	TGGGCCAATAGATGATTTTCAGAGCCTTAC			
pro-NDM-F	TTGCTCAGCTTGTTGATTATCATATGGCTT	969	61	Amplifying the *bla*$_{NDM-1}$ gene with original promoter
pro-NDM-R	TCAGCGCAGCTTGTCGGC			
LA_KPC_NDM-F	TTGCTCAGCTTGTTGATTATCATATGGCTT	9,749	60	Amplifying the long-range fragment of the *bla*$_{KPC-2}$ and *bla*$_{NDM-1}$ co-carrying region
LA_KPC_NDM-R	TGGGCCAATAGATGATTTTCAGAGCCTTAC			
KPC-Region-F	CTCAAAAGCCTCGGTGTCCTC	7,815	58	Sequencing primers spanning the *bla*$_{KPC-2}$ bearing region
KPC-Region-R	TTAGTCAGGATAAACAACAATACCCAG			
NDM-Region-F	ATGGAAACCTACAATCATACATATCGG	7,314	58	Sequencing primers spanning the *bla*$_{NDM-1}$ bearing region
NDM-Region-R	CCAGCAAATTCCCACAACGGG			
KPC-spacer	AGTTCAGCTCCAGCTCCCAG	20	60	20-nt KPC spacer for gene deletion
NDM-spacer	GATCCAGTTGAGGATCTGGG	20	56	20-nt NDM spacer for gene deletion
oriT$_{KN}$-BamHI-F	cgcggatccAGTACGGGACAAGATGTGTTTTTGG	105	60	Amplifying the *oriT* from pKPC_NDM
oriT$_{KN}$-EcoRI-R	ccggaattcATAGCTAACCTCGTTAGGGGGTG			

oriT: origin of DNA transfer.

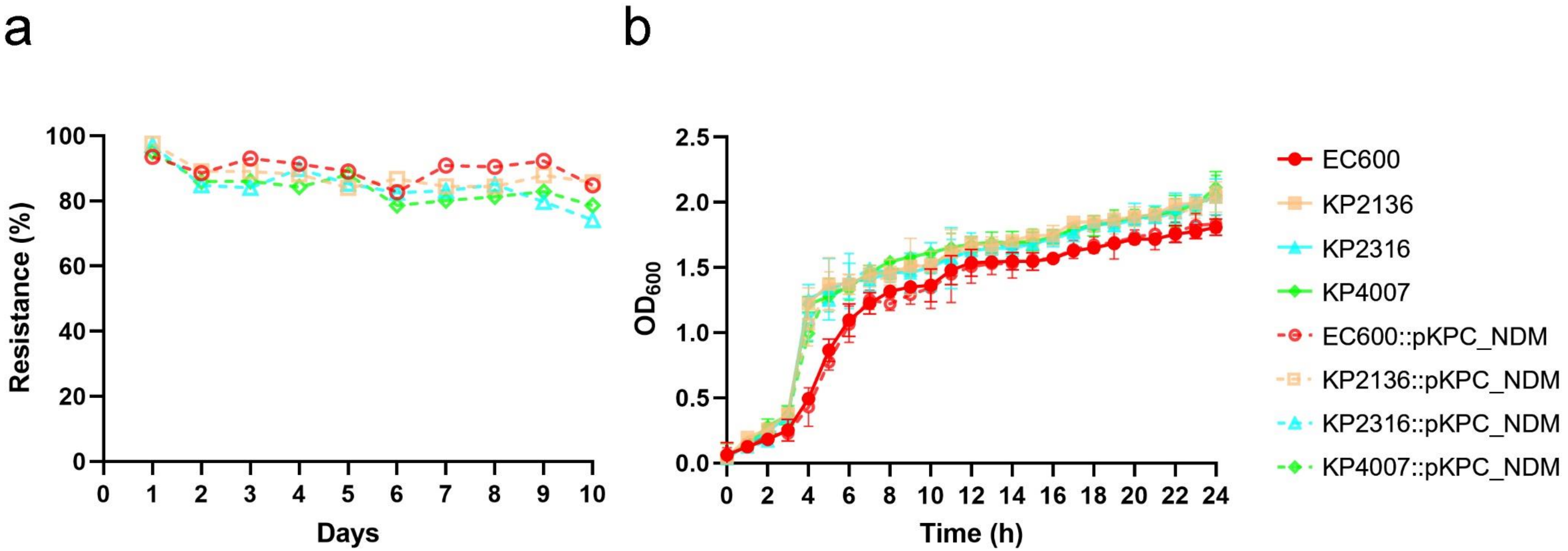

Supplementary Fig. S1 | Stability of the plasmid pKPC_NDM1527 and fitness of transconjugants. a Plasmid retention rates during 10 days of continuous passage. **b** Growth curves of the pKPC_NDM1527-carrying transconjugants.

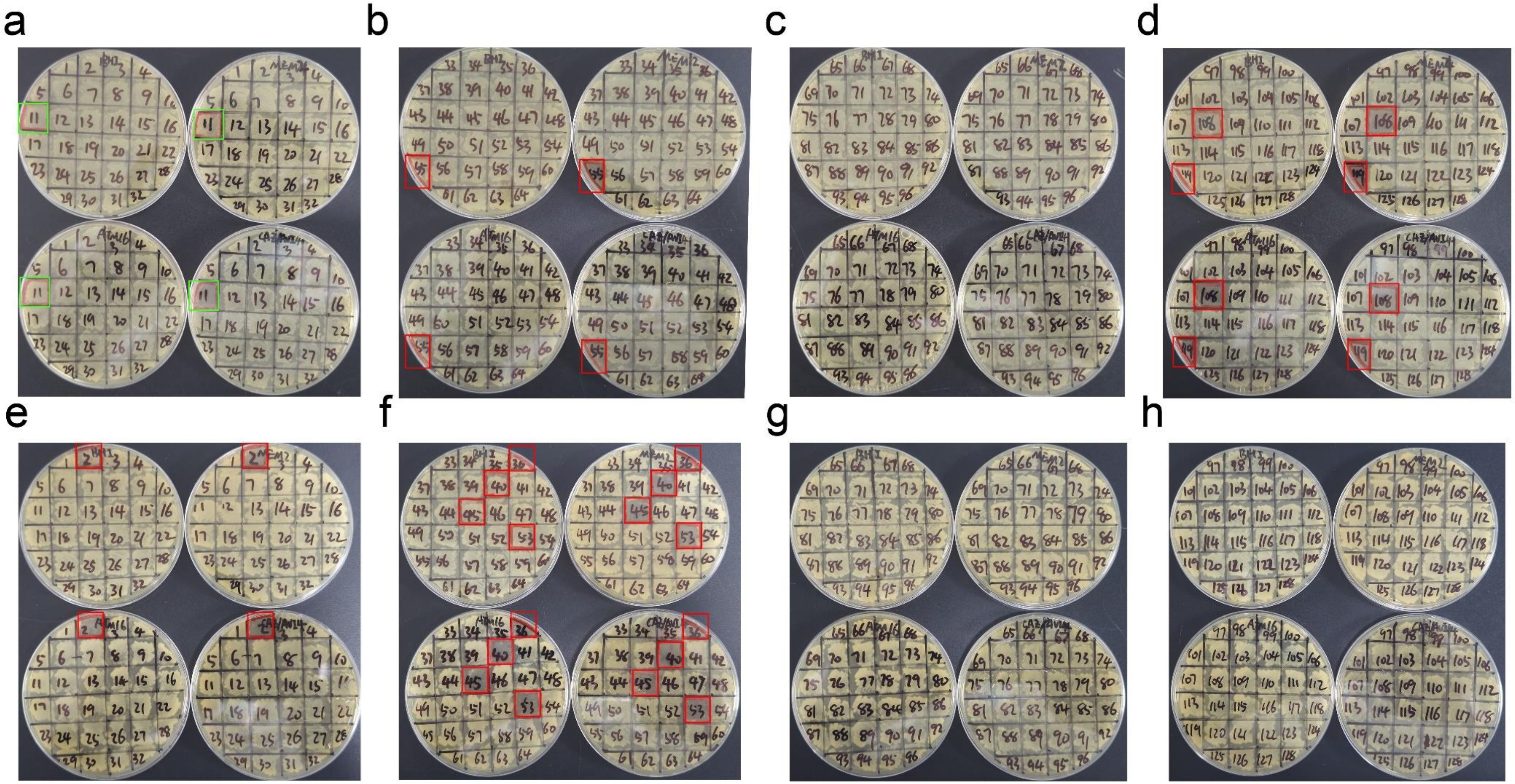

Supplementary Fig. S2 | Carbapenem resistance maintenance of transconjugants with pKPC_NDM plasmids. a–d, *E. coli* recipients, **e–h**, *K. pneumoniae* recipients. Green box: strains that lost CAZ/AVI resistance; Red box: strains that lost carbapenem resistance.

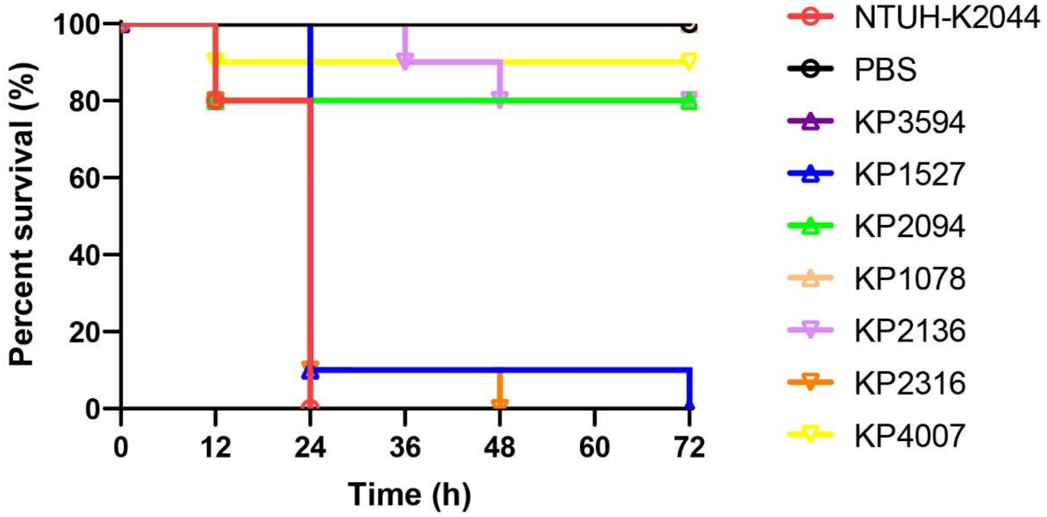

Supplementary Fig. S3 | Survival curves of the *Galleria mellonella* larvae model with ST1049 *K. pneumoniae* infections. The log-rank test was used to assess differences between survival curves.

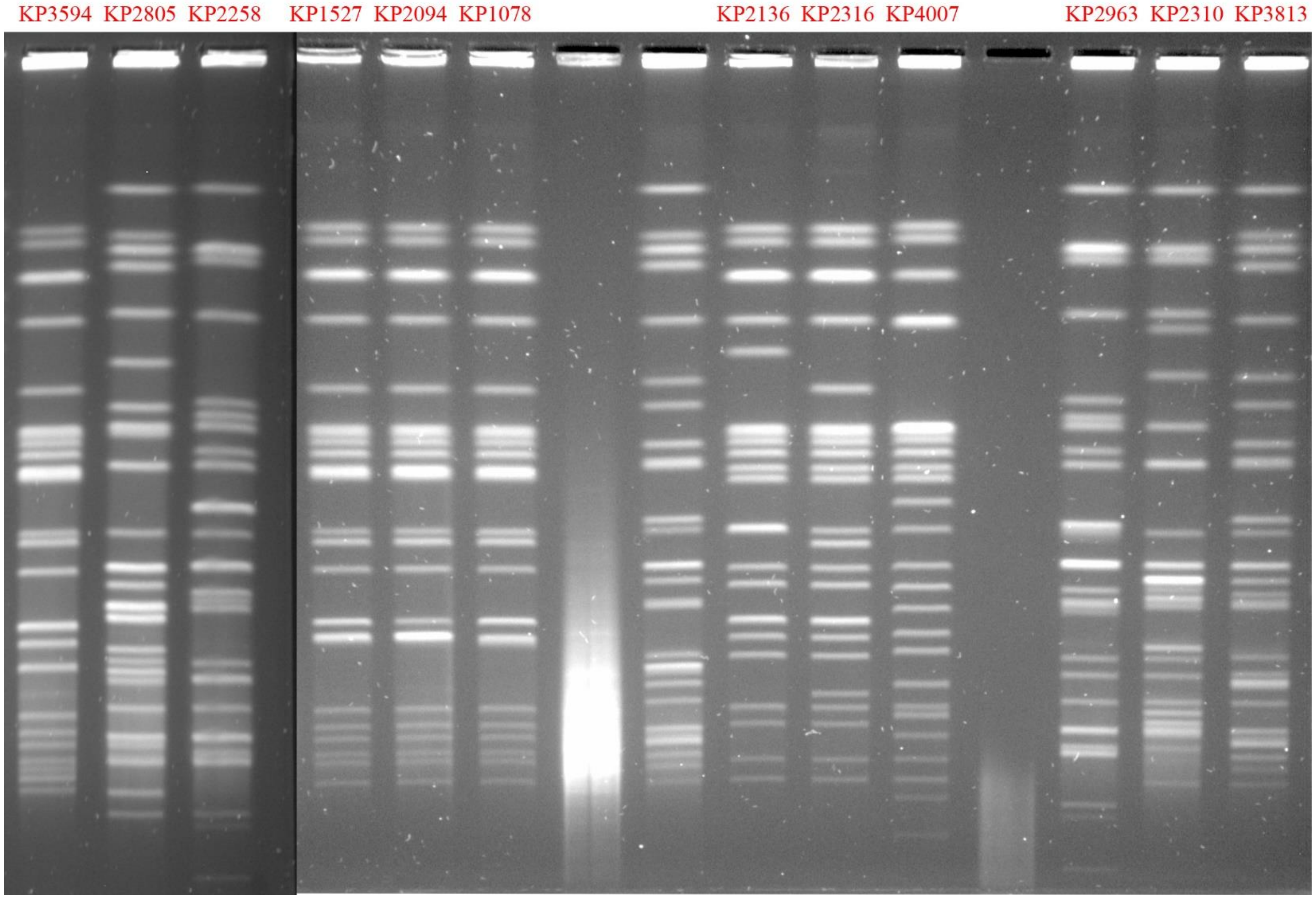

Source data: uncropped PFGE gels for Figure 1a.
