# Supplementary material for: Prevalence of ST1049-KL5 carbapenem-resistant Klebsiella pneumoniae with a blaKPC-2 and blaNDM-1 co-carrying hypertransmissible IncM1 plasmid
Source: Commun Biol. 2024 Jun 6;7:695. doi: 10.1038/s42003-024-06398-w (PMC11156905; doi:10.1038/s42003-024-06398-w)
Supplement: Supplementary file 2 — Description of additional supplementary files [file 42003_2024_6398_MOESM2_ESM.docx]

Description of Additional Supplementary Files

**File name:** Supplementary Data 1

**Description:** Source data for Figures 1 – 5.

**File name:** Supplementary Data 2

**Description:** Characteristics of transconjugants in this study.

**File name:** Supplementary Data 3

**Description:** Characteristics of plasmids with identical *oriT* to pKPC_NDM.

**File name:** Supplementary Data 4

**Description:** Characteristics of ST1049 *Klebsiella pneumoniae* strains.

**File name:** Supplementary Data 5

**Description:** Characteristics of *bla*_KPC-2_ and *bla*_NDM-1_ co-carrying *Klebsiella pneumoniae* strains
