# Supplementary material for: Prevalence of ST1049-KL5 carbapenem-resistant Klebsiella pneumoniae with a blaKPC-2 and blaNDM-1 co-carrying hypertransmissible IncM1 plasmid
Source: Commun Biol. 2024 Jun 6;7:695. doi: 10.1038/s42003-024-06398-w (PMC11156905; doi:10.1038/s42003-024-06398-w)
Supplement: Supplementary file 8 — Reporting summary [file 42003_2024_6398_MOESM8_ESM.pdf]

nature portfolio

Corresponding author(s): Yirong Li

Last updated by author(s): May 14, 2024

Reporting Summary

Nature Portfolio wishes to improve the reproducibility of the work that we publish. This form provides structure for consistency and transparency in reporting. For further information on Nature Portfolio policies, see our Editorial Policies and the Editorial Policy Checklist.

Statistics

For all statistical analyses, confirm that the following items are present in the figure legend, table legend, main text, or Methods section.

n/a	Confirmed	
☐	☒	The exact sample size (*n*) for each experimental group/condition, given as a discrete number and unit of measurement
☐	☒	A statement on whether measurements were taken from distinct samples or whether the same sample was measured repeatedly
☐	☒	The statistical test(s) used AND whether they are one- or two-sided<br>*Only common tests should be described solely by name; describe more complex techniques in the Methods section.*
☒	☐	A description of all covariates tested
☐	☒	A description of any assumptions or corrections, such as tests of normality and adjustment for multiple comparisons
☐	☒	A full description of the statistical parameters including central tendency (e.g. means) or other basic estimates (e.g. regression coefficient) AND variation (e.g. standard deviation) or associated estimates of uncertainty (e.g. confidence intervals)
☐	☒	For null hypothesis testing, the test statistic (e.g. *F*, *t*, *r*) with confidence intervals, effect sizes, degrees of freedom and *P* value noted<br>*Give P values as exact values whenever suitable.*
☒	☐	For Bayesian analysis, information on the choice of priors and Markov chain Monte Carlo settings
☒	☐	For hierarchical and complex designs, identification of the appropriate level for tests and full reporting of outcomes
☒	☐	Estimates of effect sizes (e.g. Cohen's *d*, Pearson's *r*), indicating how they were calculated

Our web collection on statistics for biologists contains articles on many of the points above.

Software and code

Policy information about availability of computer code

Data collection	Clinical information was collected retrospectively from electronic medical records. ST1049 K. pneumoniae and KPC_NDM_CRKP genomes were selected and downloaded from the PATRIC database (as of 2023-07-01), while the plasmids with the identical oriT sequences to pKPC_NDM were collected from the NCBI Nucleotide database.
Data analysis	Genome assembly was conducted with SPAdes v3.15.5 and Canu v2.2. Antimicrobial resistance (AMR) genes, virulence genes, and plasmid replicon types were identified by aligning the assembled genomes against ResFinder, VFDB, and PlasmidFinder databases in CGE services (http://www.genomicepidemiology.org/) and Kleborate v2.3.2. Phylogenetic analyses utilized core genome single nucleotide polymorphism (cgSNP) analyses performed with Snippy v4.6.0 (https://github.com/tseemann/snippy) and core genome multi-locus sequence typing (cgMLST) using chewBBACA v3.0.0. CgMLST results were visualized by the online PHYLOViZ v2.0 program. Comparative genomic analysis was performed using BLAST Ring Image Generator (BRIG) v0.95 and EasyFig v2.2.3. Elements essential for bacterial conjugation were predicted and compared against online oriTFinder (including oriTDB) and VRprofile programs.

For manuscripts utilizing custom algorithms or software that are central to the research but not yet described in published literature, software must be made available to editors and reviewers. We strongly encourage code deposition in a community repository (e.g. GitHub). See the Nature Portfolio guidelines for submitting code & software for further information.

Data

Policy information about availability of data

All manuscripts must include a data availability statement. This statement should provide the following information, where applicable:

- Accession codes, unique identifiers, or web links for publicly available datasets
- A description of any restrictions on data availability
- For clinical datasets or third party data, please ensure that the statement adheres to our policy

All data used in this study are presented in this published article and supplementary files. Genome sequencing data is publicly available in NCBI GenBank database under BioProject accession number PRJNA1019652.

Research involving human participants, their data, or biological material

Policy information about studies with human participants or human data. See also policy information about sex, gender (identity/presentation), and sexual orientation and race, ethnicity and racism.

Reporting on sex and gender	male and female
Reporting on race, ethnicity, or other socially relevant groupings	This research does not include data on race, ethnicity, or other socially relevant groupings.
Population characteristics	Patients with KPC_NDM_CRKP or ST1049 K. pneumoniae infection during the hospitalization.
Recruitment	No incentives were provided and there is no bias in selection.
Ethics oversight	All information involving human participants in this study was approved by the Medical Ethics Committee of Zhongnan Hospital of Wuhan University (2021128K)

Note that full information on the approval of the study protocol must also be provided in the manuscript.

Field-specific reporting

Please select the one below that is the best fit for your research. If you are not sure, read the appropriate sections before making your selection.

☒ Life sciences ☐ Behavioural & social sciences ☐ Ecological, evolutionary & environmental sciences

For a reference copy of the document with all sections, see nature.com/documents/nr-reporting-summary-flat.pdf

Life sciences study design

All studies must disclose on these points even when the disclosure is negative.

Sample size	This study included 4 ST1049 KPC_NDM_CRKP, 5 ST11 KPC_NDM_CRKP and 3 ST1049 K. pneumoniae isolates.
Data exclusions	No data was exclude from the analysis.
Replication	Three independent replications were perform on the growth curve, biofilm formation assay and conjugation efficiency calculations.
Randomization	Colonies were randomly selected for carbapenemase resistance maintenance and incompatibility analyses.
Blinding	Blinding was not relevant to this study because no intervention was taken with the patients.

Reporting for specific materials, systems and methods

We require information from authors about some types of materials, experimental systems and methods used in many studies. Here, indicate whether each material, system or method listed is relevant to your study. If you are not sure if a list item applies to your research, read the appropriate section before selecting a response.

Materials & experimental systems

n/a	Involved in the study	
☒	☐	Antibodies
☒	☐	Eukaryotic cell lines
☒	☐	Palaeontology and archaeology
☒	☐	Animals and other organisms
☐	☒	Clinical data
☒	☐	Dual use research of concern
☒	☐	Plants

Methods

n/a	Involved in the study	
☒	☐	ChIP-seq
☒	☐	Flow cytometry
☒	☐	MRI-based neuroimaging

Clinical data

Policy information about clinical studies

All manuscripts should comply with the ICMJE guidelines for publication of clinical research and a completed CONSORT checklist must be included with all submissions.

Clinical trial registration	Not applicable
Study protocol	Not applicable
Data collection	Clinical information was collected retrospectively from electronic medical records between September 2020 and April 2022.
Outcomes	Clinical outcomes were assessed and documented by clinical physicians according to patient's discharge summary and clinical follow-up, based on the clinical features including vital signs, biochemical indices (infection indexes), and imagine examinations on whether if the infection was controlled or underlying disease was cured.

Plants

Seed stocks	Not involved
Novel plant genotypes	Not involved
Authentication	Not involved
